# Intraplaque Hemorrhage Is a Key Finding in Sudden Cardiac Death Resulting From Coronary Vasospasm

**DOI:** 10.1016/j.jaccas.2025.103387

**Published:** 2025-03-05

**Authors:** Akihiro Takasaki, Hiromasa Ito, Tairo Kurita, Kaoru Dohi

**Affiliations:** Department of Cardiology and Nephrology, Mie University Graduate School of Medicine, Tsu, Mie, Japan

**Keywords:** acute coronary syndrome, cardiovascular disease, imaging

## Abstract

Intraplaque hemorrhage is among the most important factors associated with plaque instability and atherosclerotic progression. It is also reported to be associated with cardiac sudden death and coronary vasospasm. We report the case of a patient with cardiopulmonary arrest caused by acute myocardial infarction secondary to coronary vasospasm where intraplaque hemorrhage was observed in the culprit vessel. This is an extremely important case report that demonstrates the associations among coronary vasospasm, intraplaque hemorrhage, and sudden cardiac death with comprehensive cardiovascular imaging.

## Graphical Abstract

**Figure:**
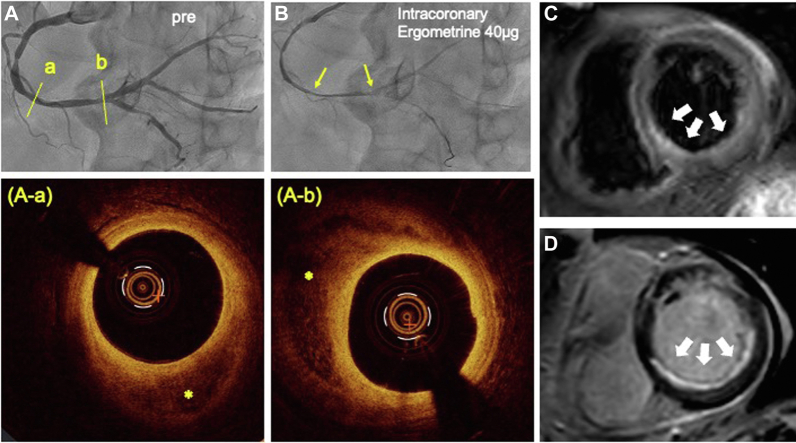

A 76-year-old man with a history of diabetes mellitus and atrial fibrillation visited his local clinic with a chief report of dyspnea. Shortly after arriving at the clinic, he collapsed, and cardiopulmonary resuscitation (CPR) was commenced, with a diagnosis of cardiac arrest. The shock was not activated by the automated external defibrillator, and the electrocardiogram (ECG) of the emergency medical service showed pulseless electrical activity. CPR was continued, and spontaneous circulation was restored in the ambulance before his arrival at our hospital (Mie University Hospital, Japan). A 12-lead ECG in the ambulance showed ST-segment elevation in leads Ⅱ, Ⅲ, and aVF, but no obvious ECG change was documented on arrival. Acute management, including hypothermic therapy, was prioritized because his hemodynamic status was stable.

**Take-Home Messages**

• This case highlights the associations among coronary vasospasm, IPH, and sudden cardiac death.
• IPH is a key finding in sudden cardiac death resulting from coronary vasospasm.

Coronary angiography was performed on day 5 of admission after his condition was fully resolved. No significant stenosis was found in the left coronary artery. The right coronary artery (RCA) also showed only moderate stenosis, but because of ST-segment elevation in ECG leads II, III, and aVF in the acute phase, intravascular imaging with optical coherence tomography (OCT) was evaluated to identify the culprit lesion (Figure 1A). OCT showed a region of low signal intensity without attenuation, highly suggestive of intraplaque hemorrhage (IPH) in multiple locations (Figures 1A-a and 1A-b), but plaque ruptures or mural thrombi were not confirmed. Subsequently, severe and diffuse spasm in the RCA was induced by an intracoronary ergonovine provocation test, consistent with the site of the IPH findings (Figure 1B). In addition, the high-intensity area on T2-weighted cardiac magnetic resonance (CMR) performed 12 days after onset and the late gadolinium enhancement area on CMR in the RCA territory indicated that the RCA was the possible corresponding culprit artery (Figures 1C and 1D).

**Figure 1:**
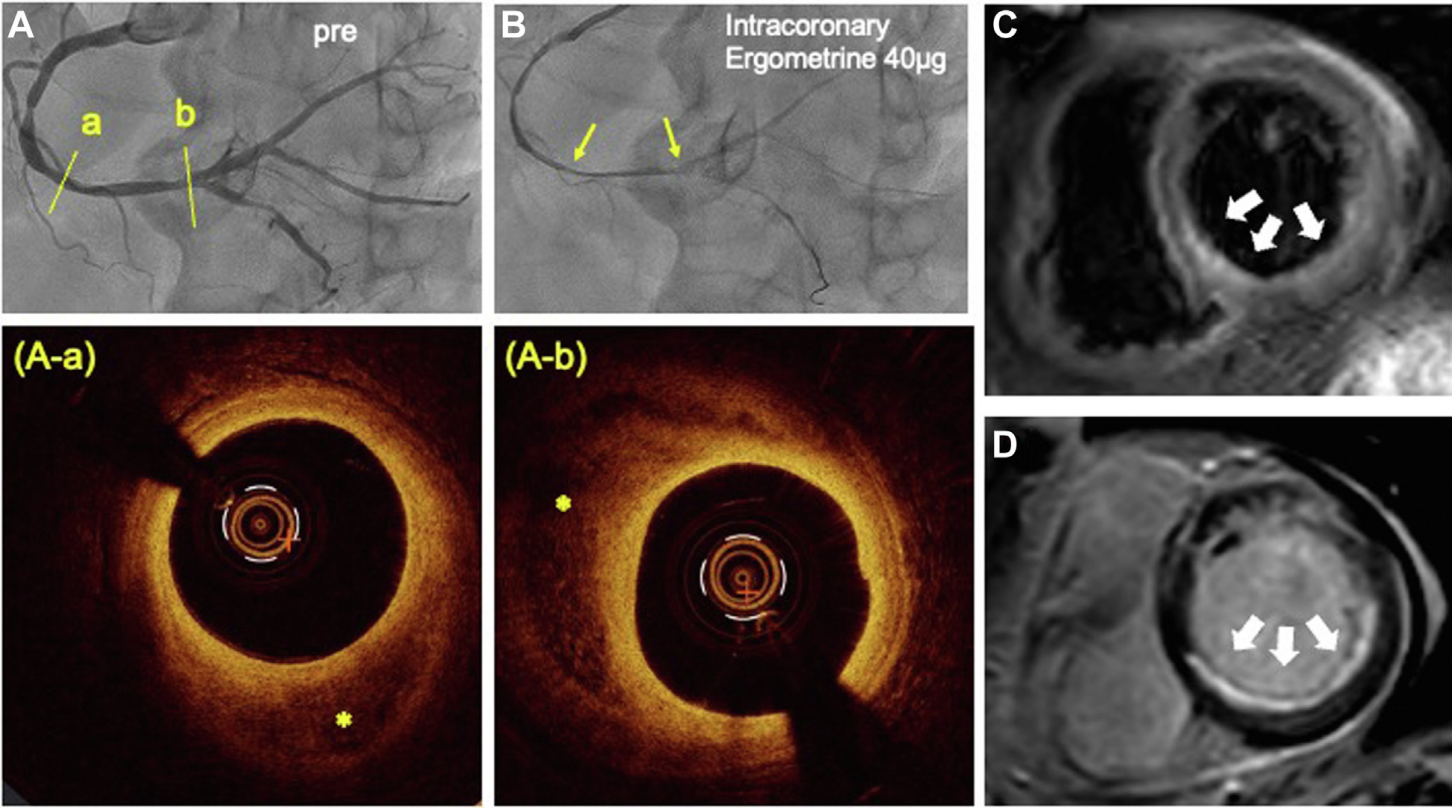
Imaging of Intraplaque Hemorrhage (A) Coronary angiogram of right coronary artery before (pre) an ergonovine provocation test. (B) Severe diffuse stenosis induced by ergometrine (40 μg) was observed in the right coronary artery (a and b, yellow arrows) (A-a and A-b) Optical coherence tomography showed a region of low signal intensity without attenuation, highly suggestive of intraplaque hemorrhage (asterisks). (C and D) Cardiac magnetic resonance showed high signal intensity (white arrows) on T2-weighted images, and subendocardial late gadolinium enhancement was observed in the territory of the right coronary artery.

Previous autopsy case reporting showed that histopathologic IPH corresponds to an OCT-detected low-intensity area without attenuation.^1^ IPH is among the most important factors associated with plaque instability and atherosclerotic progression. In addition, these findings cause endothelial dysfunction, chronic inflammation, and oxidative stress, which may trigger coronary vasospasm.^2^ Conversely, recurrent coronary vasospasm induces IPH leading to rapid plaque progression, and coronary vasospasm and IPH may form a vicious cycle.^3^ Given these considerations, it seems reasonable to consider IPH a representative finding in sudden cardiac death, as suggested by recent histopathologic studies.^4^

This is an extremely important case report that demonstrates the associations among coronary vasospasm, IPH, and sudden cardiac death with comprehensive cardiovascular imaging.

## Funding Support and Author Disclosures

The authors have reported that they have no relationships relevant to the contents of this paper to disclose.
